# Rhegmatogenous retinal detachment in uveitis

**DOI:** 10.1186/s12348-017-0140-5

**Published:** 2017-11-21

**Authors:** Joeri De Hoog, Josianne C. Ten Berge, Fahriye Groen, Aniki Rothova

**Affiliations:** Department of ophthalmology, Erasmus Medical Center, ’s-Gravendijkwal 230, 3015 CE Rotterdam, The Netherlands

## Abstract

**Background:**

Retinal detachment is more common among uveitis patients than in the general population. Here, we aimed to assess the prevalence of rhegmatogenous retinal detachment (RRD) in a uveitis population.

**Methods:**

We retrospectively studied 851 uveitis patients, recording characteristics such as uveitis duration, anatomical location, and cause; RRD occurrence; proliferative vitreoretinopathy (PVR) at presentation; surgical approach; reattachment rate; and initial and final visual acuity (VA).

**Results:**

RRD occurred in 26 patients (3.1%; 29 affected eyes) and was significantly associated with posterior uveitis (*p* < 0.001), infectious uveitis (*p* < 0.001), and male gender (*p* = 0.012). Among cases of infectious uveitis, cytomegalovirus and varicella zoster virus were most commonly associated with RRD development. RRD in non-infectious uveitis was not found to be associated with any specific uveitis entity. The rate of single-operation reattachment was 48%, and the rate of final reattachment was 83%. Mean final VA was 20/125, with 41% of eyes ultimately having a VA of less than 20/200.

**Conclusion:**

Uveitis is a risk factor for RRD development, which carries a poor prognosis.

## Background

Retinal detachment (RD) is a common complication among patients with uveitis [1]. Serous RD typically occurs in Vogt-Koyanagi-Harada disease and may also be a complication of severe scleritis, while tractional RD often develops as a complication of vitritis or chorioretinitis scarring with retinal traction. Rhegmatogenous retinal detachment (RRD) seems to predominantly occur in patients with retinitis-associated posterior uveitis [2]. The prevalence of RRD among uveitis patients is reported as 3.1%, whereas the incidence of retinal detachment in the general Dutch population is around 18 per 100,000 [3–8]. Herein, we describe the up-to-date incidence of RRD in a uveitis population as well as its associations with diverse general and ocular characteristics.

## Methods

We retrospectively studied 851 consecutive patients with uveitis (not necessarily newly diagnosed) who visited the Erasmus Medical Center Rotterdam between September 2011 and August 2013 (Table 1). The study excluded patients with serous retinal detachments (*n* = 12) and those with retinal tears but without RD (*n* = 12). This study was approved by the institutional review board (waived).

**Table 1:** General and ocular characteristics of patients with uveitis with and without rhegmatogenous retinal detachment

	Uveitis with RRD *N* = 26	Uveitis without RRD *N* = 825	*p* value^a^
Mean age at onset uveitis (years)	**46**	**43**	
Male/female ratio	**17/9**	**322/503**	**0.007**
Location			
Anterior	6 (23%)	342 (41%)	0.061
Intermediate	0	79 (10%)	0.098
Posterior	15 (58%)	183 (22%)	**< 0.001**
Panuveitis	5 (19%)	200 (24%)	0.556
Scleritis	0	21 (3%)	0.410
Infectious uveitis			
Total	14	110	**< 0.001**
HSV	1 (4%)^e^	17 (2%)	0.533
VZV^b^	3 (12%)^f^	9 (1%)	**< 0.001**
CMV^c^	6 (23%)^g^	10 (1%)	**< 0.001**
Toxoplasma	2 (8%)	23 (3%)	0.145
Presumed and definitive TB	1 (4%)	24 (3%)	0.781
Candida	1 (4%)	8 (1%)	0.158
Other^d^	0	19 (2%)	0.434
Association with systemic disease			
Sarcoidosis	3 (12%)^h^	88 (10%)	0.887
HLA-B27 associated AAU	1 (4%)	70 (8%)	0.400
JIA	1 (4%)	30 (4%)	0.955
Behçet’s disease	1 (4%)	23 (3%)	0.748
VKH	1 (4%)	6 (1%)	0.083
Other	0	101 (12%)	0.057
Established ocular entity			
Birdshot chorioretinopathy	1 (4%)	30 (4%)	0.955
Other	0	30 (4%)	0.322
Unknown	5 (19%)	337 (41%)	**0.020**

*RRD* rhegmatogenous retinal detachment, *HSV* herpes simplex virus, *VZV* varicella zoster virus, *CMV* cytomegalovirus, *TB* tuberculosis, *HLA* human leukocyte antigen, *AAU* acute anterior uveitis, *JIA* juvenile idiopathic arthritis, *VKH* Vogt-Koyanagi-Harada disease

^a^A *p* value of less than 0.05 was considered statistically significant

^b^One VZV patient had bilateral RRD

^c^Two CMV patients had bilateral involvement

^d^Includes infections with *Rubella virus*, *Treponema pallidum*, and *Borrelia burgdorferi*

^e^This patient had active uveitis presenting with RRD

^f^One of the VZV cases (bilateral) had active inflammation presenting with RRD

^g^All of the CMV cases had active uveitis presenting with RRD

^h^One case with sarcoidosis had active uveitis presenting with RRD

All patients underwent a standardized diagnostic investigation, which was tailored according to inflammation localization. This protocol included radiologic chest examination, erythrocyte sedimentation rate, blood counts, serum angiotensin-converting enzyme levels, serology for syphilis and *Borrelia*, and interferon-gamma release assay test (IGRA; QuantiFERON–TB Gold In-Tube test). Patients with anterior uveitis and panuveitis also underwent human leukocyte antigen-B27 testing. Some patients underwent additional examinations based on clinical manifestations.

Behçet’s disease and ocular sarcoidosis were diagnosed using accepted international criteria [9–11]. Briefly, definitive ocular sarcoidosis was diagnosed in patients with histological confirmation. Presumed sarcoidosis was diagnosed in patients with chest imaging suggestive of sarcoidosis and without another explanation for the uveitis, but without histological proof. A diagnosis of tuberculosis-associated uveitis required a positive culture for mycobacteria in any fluid/tissue sample, while a diagnosis of presumed ocular tuberculosis was based on a positive tuberculin skin test and/or IGRA test in the presence of otherwise unexplained uveitis. Ocular toxoplasmosis was only diagnosed when proven by intraocular fluid assessment (PCR and Goldmann-Wittmer Coefficient) [12–15]. Presumed ocular toxoplasmosis was diagnosed in cases showing typical clinical features of unilateral focal necrotizing retinitis, which are sometimes associated with typical old pigmented scars. All other specific diagnoses were based on current diagnostic criteria. Definitive anatomical classification (e.g., localization and laterality of uveitis) was performed according to the guidelines of the Standardization of Uveitis Nomenclature (SUN) Working Group [16].

Diagnoses were grouped into infectious and non-infectious diseases and into established clinical ocular syndromes, such as pars planitis and birdshot chorioretinopathy. Patients with established ocular syndromes and an identified cause or association with a systemic disorder—for example, multiple sclerosis with pars planitis or documented rubella virus infection in Fuchs heterochromic uveitis syndrome (FHUS)—were classified according to the specific cause of their uveitis.

For the population of patients with RRD and uveitis, we recorded various factors such as age; sex; uveitis laterality, location, and cause; uveitis duration before RRD; and prior intraocular surgery. Our series was treated by multiple vitreoretinal surgeons using variable surgical approaches, precluding reliable and precise classification of specific surgical techniques. Statistical analyses included the chi-square test and *t* test, performed using SPSS statistical software.

## Results

Among the 851 investigated patients with uveitis, 26 (3.1%) developed RRD in a total of 29 eyes (three patients developed bilateral RRD). Table 1 presents the general and ocular characteristics of patients with and without RRD. The mean age at uveitis onset was 46 years in the RRD subset, compared to 43 years in the non-RRD group (*p* = 0.375). The mean age at RRD onset was 48 years. While uveitis patients without RRD were more commonly female (503/825; 61%), the majority of patients in the RRD group were male (65%) (*p* = 0.012). The mean duration of uveitis prior to RRD was 34 months (range, 0–228 months). Mean follow-up duration after RRD was 111, 4 months (range, 0–560 months).

Out of 29 eyes with RRD, 10 (34%) had active uveitis within 3 months before surgery (9 with viral retinitis and 1 associated with sarcoidosis). Reliable data regarding uveitis activity were unavailable for four eyes (14%). RRD was predominantly associated with posterior uveitis (15/183 patients versus 11/642 patients, *p* < 0.001), while anterior uveitis was associated with less frequent RRD development. All three patients with bilateral RRD had uveitis of the same cause in both eyes: two patients had bilateral cytomegalovirus (CMV)-related posterior uveitis, and one had bilateral varicella zoster virus (VZV)-related posterior uveitis.

RRD patients more commonly had uveitis due to infectious causes (14/26 versus 110/825; *p* < 0.001). Within the group of patients having infectious uveitis, CMV retinitis was predominantly associated with RRD development (8/29 eyes, 28%) followed by VZV (4/29 eyes, 14%) (*p* < 0.001 for both).

Of the 29 eyes with RRD, 9 (31%) had undergone previous intraocular surgery, comprising four cataract extractions, two phaco-vitrectomies (for macular puckers), one vitrectomy alone (for floaters), one trabeculectomy alone, and one trabeculectomy that was followed by vitrectomy (for floaters). Of the six eyes with RRD in the anterior uveitis group, three had previously undergone uncomplicated intraocular surgery, one of which was also highly myopic (~S−10).

Proliferative vitreoretinopathy (PVR) was present at RRD presentation in 7/29 eyes (24%). Three of these eyes exhibited PVR both before and after surgery, whereas four eyes had developed new PVR after initial surgery.

The mean number of surgical procedures for uveitic RRD was 1.6. Single-operation reattachment was achieved in 14/29 eyes (48%). In 24/29 (83%) eyes, the retina was reattached after additional surgical procedures. In 4/29 eyes (14%), the silicone oil could not be removed due to hypotony or persistent detachment. One eye did not undergo surgery due to a very poor prognosis.

The mean best corrected visual acuity (BCVA) was 20/32 before RRD and was 20/80 in eyes with RRD just prior to surgery. The mean final VA was 20/125. Of the 29 eyes with RRD, 12 (41%) ultimately had a VA of less than 20/200, including 3 eyes with only hand movements or light perception, and 4 eyes with no light perception. There was no significant difference in visual outcome between patients with PVR prior to surgery and those without, nor between viral versus non-viral causes of uveitis.

## Discussion

In our series, RRD occurred in 3.1% of patients with uveitis, constituting a serious uveitis complication that was responsible for a final VA of ≤ 20/200 in 12 (41%) of the 29 eyes affected with uveitic RRD.

The 3.1% prevalence of RRD within our cohort of patients with uveitis is similar to the prevalence reported in patients with uveitis in 2003 and is much higher than the prevalence in the general population. Moreover, 12% of patients with uveitic RRD in our study showed bilateral involvement. This is substantially higher than the reported 1.67% rate of bilateral RRD in the non-uveitis population [3, 4]. Tomkins-Netzer et al. [1] described severe vision loss (≤ 20/200) due to RRD in 1.33% of 1076 uveitis patients, which is very similar to the rate in our present series (1.4%; 12/852). Additionally, the visual outcomes among patients with RRD and uveitis were worse (average 20/125) compared to outcomes in patients with non-uveitic RRD. In cases of RRD without uveitis, the mean BCVA after surgery (with macula on and off) was between 0.17 and 0.18 logMAR, corresponding to a Snellen VA of 20/30 [17, 18], which is much better than the outcomes in our present cohort.

Our study was performed during an era of transition from 20-gauge to 23-gauge and eventually 25-gauge vitrectomy. We had incomplete data regarding the specific surgical approaches used, such as vitrectomy with or without buckle and/or encircling element or buckling surgery only, and the specific types of tamponades employed. Thus, it is not possible to determine the most efficacious surgical approach based on our data. The poor visual outcome of RRD in our series of patients with uveitis might have been partly influenced by severe co-morbidity with sometimes already compromised macular function, ongoing intraocular inflammation, and possible hypotony. In the future, the continuous evolution of vitreous surgery might reduce the visual impairment in uveitis patients who develop RRD. Our data highlight the need for additional investigations, and preferably prospective studies, of the best medical and surgical approaches for RRD in uveitis.

Our results indicated that uveitic RRD was associated with a posterior location of uveitis and with infectious causes, especially viral infections. It is logical that retinal involvement would be related to RRD development. Retinal necrosis and atrophy related to viral retinal disorders, in combination with vitreous degeneration, fibrotic changes, and traction encountered in uveitis, likely all play a role in the development of retinal tears and subsequent detachment [19]. This may also explain why all bilateral RRDs occurred in patients with viral retinitis. In non-infectious disorders, large areas of retinal atrophy are not common; however, the higher rates of vitreous shrinkage and posterior vitreous detachments in uveitis may also contribute to RRD development.

Cytomegalovirus is a well-known cause of RRD. Notably, in the AIDS era, one third of patients with CMV retinitis developed RRD. In our series, two patients with CMV retinitis and RRD were HIV-infected and had low CD4+ leukocyte counts. The other four patients with CMV retinitis and RRD were receiving immune suppressive therapy for idiopathic thrombocytopenic purpura, combined with a splenectomy (*n* = 1), malignant T-cell lymphoma (*n* = 1), and after organ transplantation (*n* = 2). The current literature does not describe mechanisms that would explain higher RRD rates based solely on the presence of anterior uveitis. In our study, six patients with anterior uveitis suffered from RRD, and none of the above-described risk factors for RRD development seem to apply in these cases. Among these patients, three had undergone uncomplicated intraocular surgery prior to RRD onset, with intervals of 11 months, 1 year, and 5 years between surgery and RRD, which may have contributed to RRD development.

The present study had several limitations, including its retrospective design. We were unable to adequately assess the prevalence of general risk factors for RRD, such as high myopia or lattice degeneration, or the exact stage of PVR before and after surgery. Furthermore, we could not reliably determine the uveitis activity (including CME presence) at the time of RRD onset in all cases. Thus, it was impossible to estimate the number of eyes that exhibited newly developed CME after surgery. Despite these shortcomings, our results clearly document the high RRD prevalence in a uveitic population, specifically among patients with posterior and viral uveitis, as well as highlight a poorer visual outcome of RRD treatment in patients with uveitis. The only other study of RRD in uveitis was published two decades earlier and reports a similar RRD prevalence. This lack of change is surprising, as the diagnostic tools and treatment for uveitis have improved over this time period, particularly with the introduction of various novel treatment options such as anti-TNF drugs and other medications.

In conclusion, our study demonstrates a 3.1% prevalence of RRD in the general population of patients with uveitis from a tertiary center. Our results further highlight the poor prognosis of uveitic RRD and the need for improved management of such cases.
